# Influence of the Oxide and Ethanol Surface Layer on Phase Transformation of Al-Based Nanocomposite Powders under High-Energy Milling

**DOI:** 10.3390/ma12081305

**Published:** 2019-04-21

**Authors:** Dora Janovszky

**Affiliations:** MTA-ME Materials Science Research Group, Miskolc, Hungary; fekjd@uni-miskolc.hu

**Keywords:** high-energy milling, Al-based composite, process control agent, amorphous-nanostructure alloy, dry and wet grinding

## Abstract

Pure Al particles reinforced with amorphous-nanocrystalline Cu_36_Zr_48_Ag_8_Al_8_ particles composite powders were prepared by high-energy milling without and with ethanol. The mechanical milling procedures were compared so that in the case of dry milling the particle size increased owing to cold welding, but the crystallite size decreased below 113 nm. The amorphous phase disappeared and was not developed until 30 h of milling time. Using ethanol as a process control agent, the particle size did not increase, while the amorphous fraction increased to 15 wt.%. Ethanol decomposed to carbon dioxide, water, and ethane. Its addition was necessary to increase the amount of the amorphous structure.

## 1. Introduction

The demand for Al-based metal matrix composites (AMMCs) will continue increasing at unstoppable rates due to development in the automotive and construction industry. Improvement in the strength of aluminum alloys can be achieved by uniformly distributed hard materials such as oxides [1,2], borides [3], carbides [4], graphene [5,6] or carbon nanotubes [7,8]. However, wetting problems arise and microparticles severely degrade the plasticity and machinability of metals [9]. The introduction of metallic particles, particularly amorphous-nanocrystalline alloy, can solve these problems owing to their high strength, large elastic limit, high corrosion resistance, and good wettability by the metal matrix [10,11]. The powder metallurgical route can be successfully employed to fabricate an amorphous/nanocrystalline composite powders in which the reinforcing particles are uniformly distributed in the Al- matrix. In the case when one mills two different metallic powders, several solid phase transformations take place [12,13,14,15]. A process control agent (PCA) such as stearic acid, benzene, methanol, or ethanol is added to the powder mixture during milling to reduce the effect of cold welding between powder particles [16,17,18]. The PCA adsorbs on the surface of the powder particles and minimizes cold welding between powder particles and thereby inhibits agglomeration [19,20]. Furthermore, the PCA has a significant effect on the milling process [21,22]. Nowadays, the exact mechanism of different PCAs during high-energy milling and their influence on the outcome is not known. It is also unclear what organic additive is the best for each material. A large variety of PCAs was used in mechanical alloying experiments. In some Fe containing powder milling, it was found that the presence of organic PCA, except ethanol, was necessary for the formation of the amorphous phase. This fact was explained by the dissolution of C atoms from PCA in the Fe-B phase by mechanical milling [18,23]. The role of ethanol in the formation of amorphous structure is not clear during high-energy milling of the Al matrix powders either.

In this work, the influence of oxygen and ethanol on the outcome of the high-energy milling process of an Al-based composite is presented. Amorphous-nanocrystalline Cu_36_Zr_48_Ag_8_Al_8_ alloy was chosen as a reinforcing component in the form of powder. The high-energy milling process was investigated with and without ethanol as the PCA.

## 2. Material and Methods

Centrifugal cast rods with the atomic composition Cu_36_Zr_48_Ag_8_Al_8_ (the numbers indicate at. %) were ground and fractionated to a particle size below 320 μm for ball-milling. The amorphous content of the ground powder was approximately 12 wt.% based on X-ray diffraction (XRD, Bruker Gmbh, Berlin, Germany) analysis. Pure Al powder (99.5 wt.% and 15 μm average particle size, 664 °C melting point) was selected as the matrix material. Al powder with 10 wt.% Cu_36_Zr_48_Ag_8_Al_8_ particles was milled for 30 h. The mixed powder was milled at room temperature in a Pulverisette 5 high-energy ball-mill at 200 rpm under a protective argon atmosphere using a stainless-steel grinding bowl and steel balls of different diameters, which were used together. The diameters of the hardened balls were 20 mm, 12 mm, and 10 mm. Two series of experiments were carried out. In the first experiment, the weight ratio of the ball to the powder (BPR) was 10:1 (A1 experiment). To study the effect of the milling time, samples were taken between 5–30 h by interrupting the milling cycle in the case of the first milling experiment. A sample of 0.7 g was taken every 5 h to check the effect of the milling time on the particle size, shape, and amorphous content. In the second experiment (A2) the milling bowl was not opened until 30 h of milling time and some ethanol was used as the PCA. In order to avoid overheating in the vials, the milling procedure was interrupted every 60 min and halted for 60 min in both experiments.

The microstructure of the powders was examined by a Hitachi S-4800 Scanning Electron Microscope (SEM, Hitachi, Tokyo, Japan) equipped with a BRUKER AXS type energy-dispersive X-ray spectrometer (EDS). Backscattered electron micrographs were recorded in order to get information about the microstructure of the samples. The particle size distribution of the ground material was determined by a Horiba LA-950 V2 type laser diffraction particle size analyzer (Horiba Ltd., Kyoto, Japan) in distilled water. During the measurement process, 1-minute ultrasonic treatment and 1 mL of 50 g/L sodium pyrophosphate dispersant were applied to achieve the appropriate dispersity state.

Thermal analysis was performed in a Netzsch 204 DSC (Netzsch Ltd., Selb, Germany) at a heating rate of 0.66 K/s under a flow of purified argon. X-ray diffraction (XRD) phase analysis was performed by a Bruker D8 Advance diffractometer (XRD, Bruker Gmbh, Berlin, Germany) using Cu Kα radiation (40 kV, 40 mA), in parallel beam geometry obtained with a Göbel mirror equipped with a Vantec-1 position sensitive detector (1° window opening), measured in the 2–100° (2θ) angular range, at 0.007° (2θ)/29 s speed. The specimen rotated in the sample plane during the measurement to obtain data from the whole surface and to reduce in-plane preferred orientation effects. The crystalline fraction was determined by XRD analysis using peak area determination in TOPAS4 (amorphous hump method). Quantitative results were obtained by combined use of Rietveld refinement and peak area calculation [24,25]. The volatile substances in the milling bowl were analyzed by an Agilent 7890A gas chromatograph (Agilent Technologies, Inc. Wilmington, USA) equipped with Agilent 5975C mass selective detector (Agilent Technologies, Inc., Wilmington, NC, USA) and MSD ChemStation software.

## 3. Results and Discussion

### 3.1. Raw Materials

**Figure 1:**
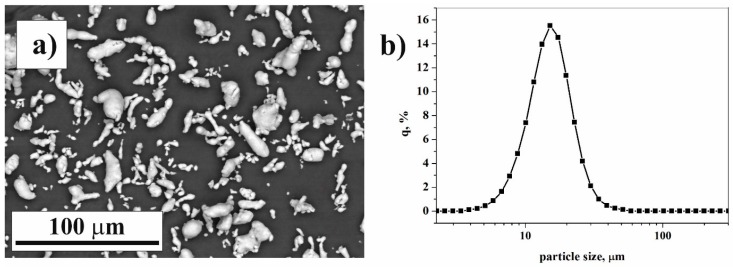
Scanning electron microscopic (SEM) image (**a**) and particle size distribution of the Al powder (**b**).

**Figure 2:**
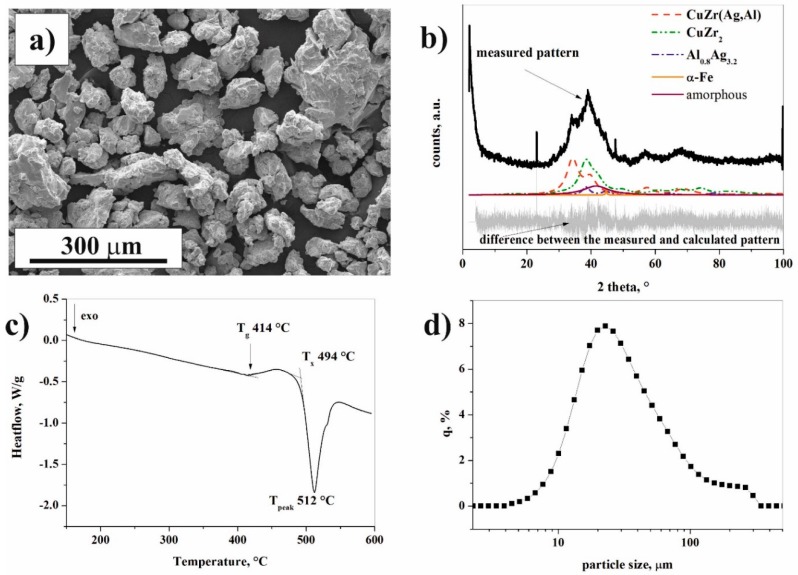
Scanning electron microscopic (SEM) images (**a**), XRD pattern (**b**), DSC thermogram (**c**), and particle size distribution (**d**) of the Cu_36_Zr_48_Ag8Al_8_ powder.

The morphology and particle size distribution of pure Al powder is shown in Figure 1. The particle size of the irregular Al alloy powder, as measured by a laser diffraction particle size analyzer, spans in the range of 4–51 µm (Figure 1b). The mode of Al powder is 14.2 µm. Brittle fracturing can be observed on the surface of ground Cu_36_Zr_48_Ag_8_Al_8_ powder (Figure 2a). The X-ray diffraction pattern of ground Cu_36_Zr_48_Ag_8_Al_8_ powder presents sharp Bragg peaks superimposed on the halo due to the presence of an amorphous phase (Figure 2b). Four crystalline phases were identified: CuZr (Fm-3m, 8.4 wt.%), CuZr_2_ (I4/mmm, 80.3 wt.%), Al_0.8_Ag_3.2_ (Fm-3m, 9.6 wt.%) and α-Fe (Fm-3m, 1.7 wt.%). The crystallite sizes are presented in Table 1. The unit cell of the CuZr phase is a = 0.3262 nm [26]. However, the Rietveld refinement shows that the unit cell of the CuZr phase is a= 0.4541 nm, i.e., the crystal structure of this phase is distorted. This phenomenon occurs when Cu atoms are replaced by Ag and Al atoms, which have smaller atomic radii (r_Al_ = 0.143 nm, r_Ag_ = 0.144 nm) compared to Cu (r_Cu_ = 0.128 nm). This phase is hereinafter referred to as the CuZr(Ag,Al) phase based on the XRD results. The “X-ray amorphous structure” content is 11.2 wt. %. Observing the amorphous halo, the maximum position of the halo is 0.2182 nm, which reflects the most frequent distances between atoms [27]. Figure 2c shows the thermal properties of Cu_36_Zr_48_Ag_8_Al_8_ amorphous-nanocrystalline powder. The glass temperature (T_g_) is 414 °C, and the onset of crystallization (T_x_) is 494 °C. The mode of the Cu_36_Zr_48_Ag_8_Al_8_ particle size distribution by LPSA is 21.3 µm (Figure 2d). It is noteworthy that a small fraction of larger particles can be detected.

The amorphous content of raw Cu_36_Zr_48_Ag_8_Al_8_ alloy is characterized by two halos in the XRD pattern, which indicates the existence of two short-range orders in the amorphous phase. The first peak position of the amorphous halo is 0.22781 nm, and the second peak position is 0.13896 nm. The first short-range order is probably enriched in Zr, while the second one is enriched in Cu. Both the short-range orders contain Ag and Al atoms, too.

### 3.2. Composite Material of A1 Experiment (without Ethanol)

#### 3.2.1. Particle Morphology and Size Distribution

**Figure 3:**
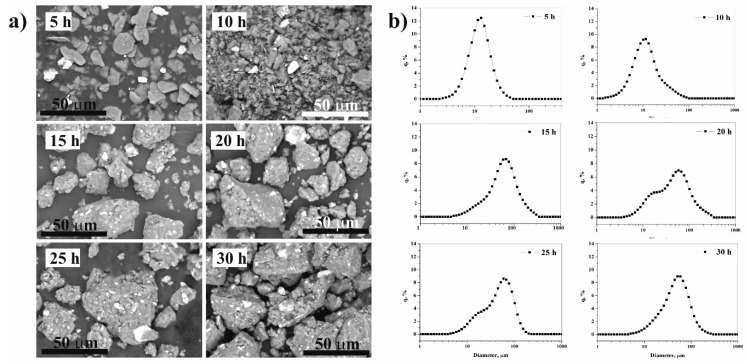
Backscattered SEM images (**a**) and particle size distribution (**b**) of mixed powders milled for different periods of time.

**Figure 4:**
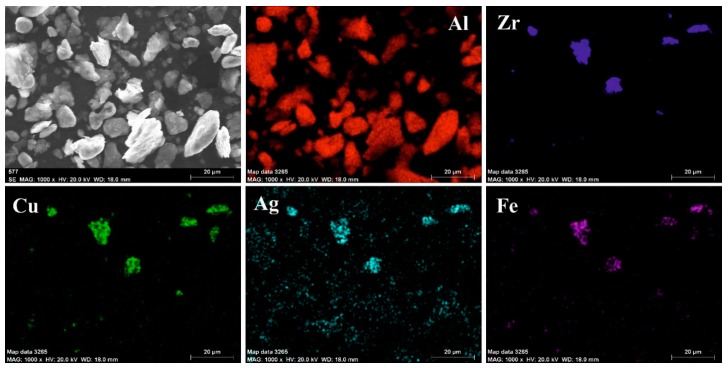
SEM micrograph and characteristic compositional XEDS-SEM mapping of 5 h milled powder.

Figure 3a shows the morphological variation of Al-CuZrAgAl composition obtained by SEM after 5 h, 10 h, 15 h, 20 h, 25 h, and 30 h milling time, respectively. The darker particles in all the backscattered SEM images correspond to Al particles, whereas the brighter phase indicates the Cu_36_Zr_48_Ag_8_Al_8_ particles. At the 5-hour milling stage, the ductile Al powder particles are flattened, plastically deformed and fractured due to the collision of the grinding balls. The Cu_36_Zr_48_Ag_8_Al_8_ particles are fragmented and these particles are separated from each other and from the Al particles (Figure 3a). The particle size distribution is symmetric (Figure 3b). The mode of the composite particle is 12.3 μm. The α-Fe content is considered contamination of the milling process coming from the milling media. Based on the XEDS-SEM mapping (Figure 4), the α-Fe can be detected on the surface of Cu_36_Zr_48_Ag_8_Al_8_ particles already after 5 h milling time.

After 10 h milling time, the fragmentation of the Al particles is remarkable (Figure 3a). It is quite clear from all SEM images that besides the fragmentation, the agglomeration of the Al particles takes place. The particle size distribution confirms this process; larger particles appear (Figure 3b), and the distribution curve is no longer symmetrical. The bigger Cu_36_Zr_48_Ag_8_Al_8_ particles are not yet generally connected to the Al particles.

**Figure 5:**
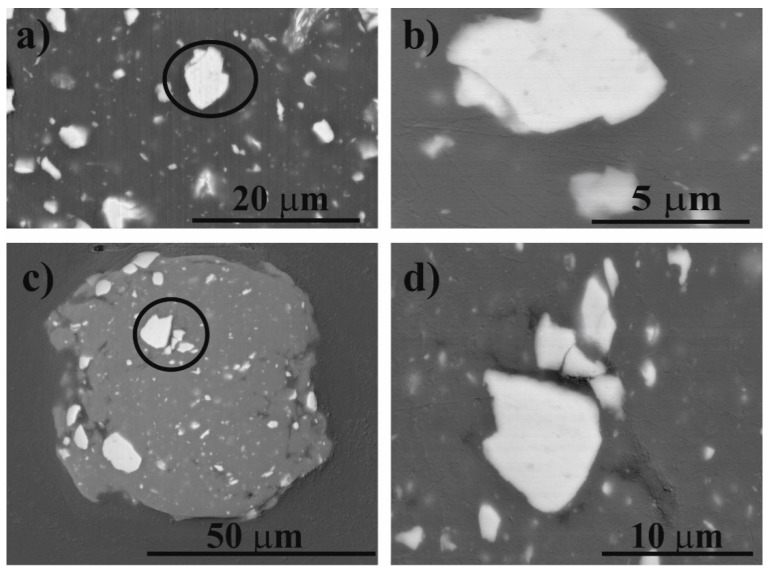
Cross-sectional backscattered SEM images of composite particles after 15 h (**a**,**b**) and 25 h (**c**,**d**) of milling time. Representative images showing particle/matrix interface (**b**,**d**).

The most significant change is observed between 10 and 15 h milling time. This could be attributed to the tendency that cold welding between the Al-Al and Al-Cu_36_Zr_48_Ag_8_Al_8_ particles dominates their fracture. After 15 h of milling, the mode of composite powder has increased to 63.0 μm. Al particles are stuck to and surround the reinforcing particles (Figure 3a). Further milling results in severe plastic deformation of clusters and reduction of the particle size. With the milling time extended, composite powders are broken up and cold welding repeatedly takes place. The volume distribution by LPSA shows that bimodal distribution is formed after 20 and 25 h of milling time (Figure 3b). The higher mode of particles decreases to 55.1 μm and 55.3 μm after 20 and 25 h milling time, respectively. The smaller mode of particles is 22.8 μm in both cases. This bimodal character disappears after further milling. The mode of particles decreases to 48.5 μm after 30 h milling time (Figure 3b). The cross-sectional SEM micrograph of the composite particles shows good bonding at the matrix/particle interface (Figure 5). Many reinforcing particles are trapped in the cold-welded Al particles, which is the matrix. The reinforcing particles are fractured into several pieces owing to the large shear deformation. Some broken pieces of reinforcing particles can be seen in the inside composite particle (Figure 5c,d) or/and on the surface of the composite particles (Figure 5c).

**Figure 6:**
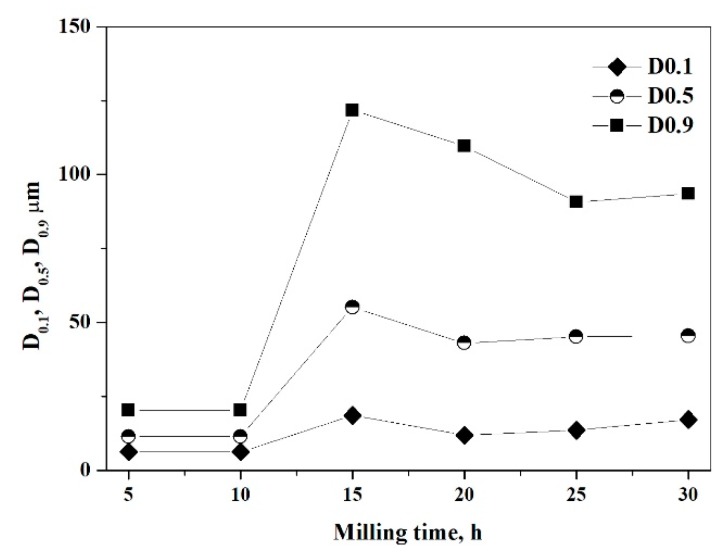
Particle sizes D_0.1_, D_0.5_, and D_0.9_ of the composite as a function of the milling time (D_0.1_, D_0.5_, and D_0.9_ diameters correspond to 10, 50, and 90% fineness of the particle size volume distribution, respectively).

Summarizing the morphology and particle size analysis, we can conclude that in the initial stage (5 h milling time), the ductile Al phase is flattened, and the brittle phase is fragmented. Further milling leads to the formation of new surfaces between the fractured and deformed powder particles, which is due to the force of impact. These new surfaces cold weld and larger particles form due to the large surface area and the lack of surfactant to prevent direct contact between them. Then the Al-Al and Al-reinforcing phases would stick together, which causes the average powder size to increase after 15 h of milling (Figure 6).

With the milling time extended, the ductile Al particles become harder due to the cold work of the balls. The composite powder is repeatedly broken and cold-welded. Between 15 and 20 h of milling time, fracturing is the determining factor that causes reduction in the particle size. Observing the median of the particle size distribution, a steady state is reached after 25 h milling time.

#### 3.2.2. Phases and Grain Size Analyses using the XRD Method

**Figure 7:**
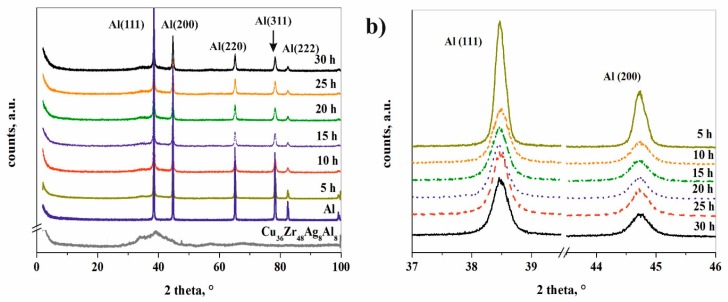
XRD patterns of the composite powders and initial powders (**a**) and intensity reduction of Al (111) and (200) peaks at different time intervals (**b**).

Figure 7a shows the XRD patterns of Al-composites with different milling times and of the initial powders. The pattern of composite powder after ball milling for 5 h displays sharp Al peaks with other peaks but without an amorphous halo. During the milling process, crystalline defects (dislocations and stacking faults) form due to the repeated impact and shear forces of the milling media [26,28]. Owing to the input energy of the milling process, sub-grains, and high-angle grain boundaries are created in the Al matrix. Significant broadening and height reduction in the intensity of the Al peaks are observed with milling time owing to grain size refinement, lattice strain (Figure 7b). The increase in the width of XRD peaks clearly indicates the grain size reduction and increase in the lattice strain (Table 1).

It is well known in the literature that oxygen has an essential effect on the glass forming ability [29,30]. Furthermore, the interface between an amorphous particle and its oxide layer can act as a heterogeneous nucleation site for crystallization during thermal annealing of the amorphous particle [31]. A thin oxide layer exists on the surface of both initial powders. If CuZr(Ag,Al), CuZr_2_ or amorphous phases are located on a particle surface, the composition of the layer is ZrO_2_ [32]. In the case of the Al and Al_0.8_Ag_3.2_ phase, the oxide layer is Al_2_O_3_. Under the milling process of the reinforcing particles Cu, Al, Ag and Zr atoms become surface atoms. Of course, these atoms can also be oxidized by the atmosphere. Despite using argon gas during the milling process, oxygen is found in the milling material, and one can only minimize further oxidation.

**Figure 8:**
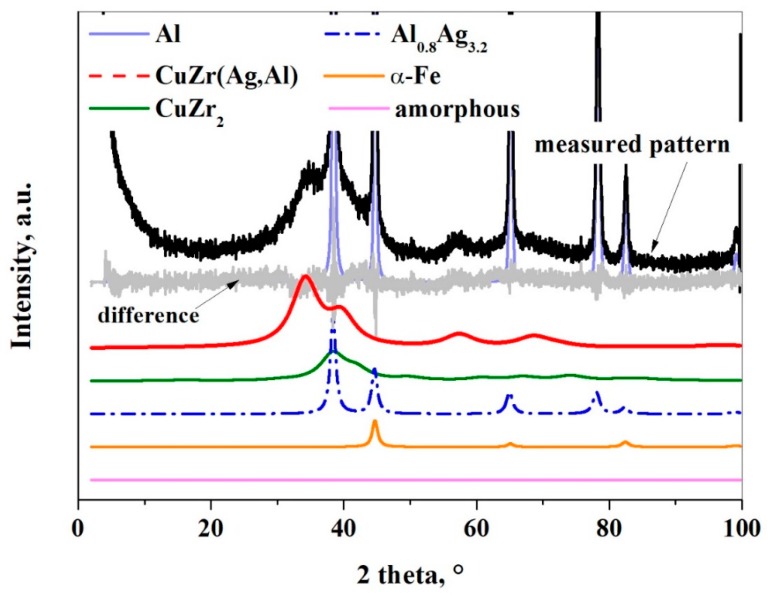
XRD pattern of the composite powder after 30 h milling time without using ethanol.

According to the Rietveld refinement, the initial amorphous-nanocrystalline Cu_36_Zr_48_Ag_8_Al_8_ alloy transforms into nanocrystalline phases after 5 h milling time (Table 1). It can be established that the thin oxide layer has such a strong effect that it prevents the amorphization despite the high amount of input energy during the milling process. Three phases crystallize from the amorphous-nanocrystalline alloy due to the input energy from milling: Al_0.8_Ag_3.2_, CuZr_2_, and CuZr(Ag,Al) (Figure 8 and Table 1). J. Cui et al. [33] investigated the crystallization of Cu_36_Zr_48_Ag_8_Al_8_ alloy in non-isothermal and isothermal modes. Under non-isothermal conditions, the CuZr phase and the AlAg phase crystallize, while under isothermal conditions the CuZr, AlAg and AgZr phases crystallize [33]. It is worth noting that CuZr_2_, AlCu_2_Zr, AgZr_2_, and unknown phases were identified by Wei Zhang et al. [34] in water-quenched Cu_36_Zr_48_Ag_8_Al_8_ alloy with a 25 mm diameter. It can be stated that different phases crystallized partly due to the milling process rather than the solidifying process.

**Figure 9:**
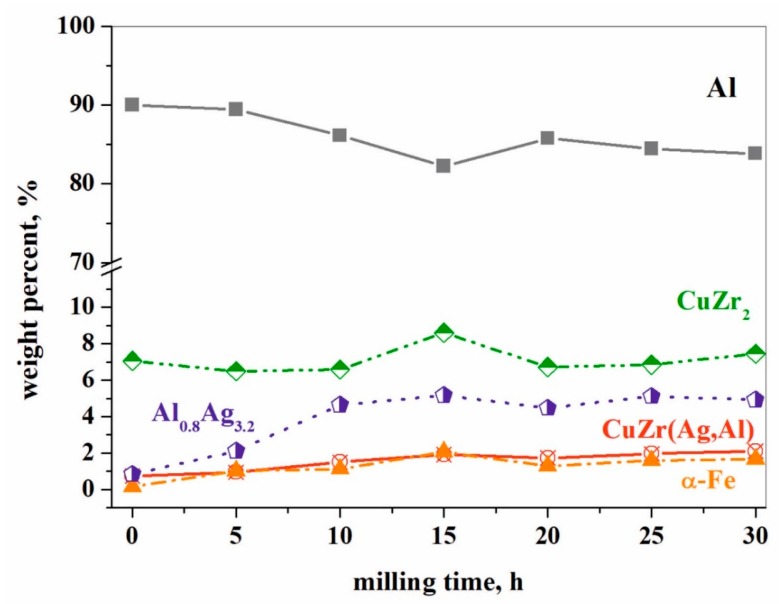
Effect of milling time on the weight fraction of different phases.

**Figure 10:**
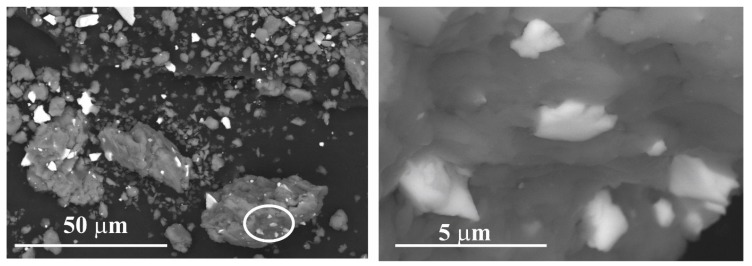
Backscattered SEM images of the composite material after 30 h milling time using ethanol as PCA (**a**) and magnified image of the area marked by the circle (**b**).

The weight fraction of the CuZr_2_ phase is approximately constant during the whole milling process (Figure 10a). On the contrary, the amount of the aluminum-containing phases strongly increases; the weight fractions of the Al_0.8_Ag_3.2_ and CuZr(Ag,Al) phases increase by more than 5 and 2 times, respectively, at the end of milling process (Table 1). The impurity α-Fe content increases with the milling time up to 15 h (Figure 9). The maximal content that evolved in our experiments was 2.1 wt. % according to XRD.

The crystallite size of the Al matrix and other phases in the mechanically milled powders can be calculated by the Rietveld refinement. After 5 h of milling time, the crystallite size of CuZr_2_ and CuZr(Ag,Al) is 1.27–2.0 nm and is constant during further milling (Table 1). On the contrary, the crystallite size of Al powder decreases drastically from 494–776 nm to 50–79 nm within 10 h of milling. Between 15 and 30 h of milling time, the change of crystallite size is not significant. The crystallite size of the Al_0.8_Ag_3.2_ phase increases after 5 h milling time and decreases to the near initial size after 10 h of milling. With further milling, the crystallite size remains approximately constant.

### 3.3. Composite Material of the A2 Experiment (with Ethanol)

In the case of the A2 experiment, the grinding bowl is not opened during 30 h of milling. Before milling the atmosphere is filled with argon and some ethanol as a process control agent (PCA) is added. With every opening, the air inevitably enters the grinding bowl. It is known that ethanol adsorbs on the powder surface and reduces consequently the surface tension of the solid material [28]. The molecule size of ethanol is 0.44 nm. Ethanol is a good wetting agent for all metallic materials. Ethanol fills first the places where the surface tension is greater, that is, the gaps and the pores of metallic particles. Later it covers the surface of particles where the surface tension is smaller. Copper oxide removal can be achieved by ethanol at temperatures as low as 130 °C [35]. The reduction of silver oxide by ethanol can also take place (silver mirror test).

One can observe a significant difference between wet milled and dry milled samples. The presence of ethanol diminishes the cold welding between Al-Al particles and Al-reinforcing particles. SEM micrograph of the composite powder reveals that many of the Cu_36_Zr_48_Ag_8_Al_8_ alloy particles are not cold-welded to Al particles (Figure 10a). Due to the intensive impacts of the balls—as a result of collision—the ethanol and oxide layers break. However, not only the thin layer breaks but the particles also. New active surfaces develop so cold welding between the particles can take place. Figure 10b shows that the small Cu_36_Zr_48_Ag_8_Al_8_ alloy particles are cold-welded to Al particles.

**Figure 11:**
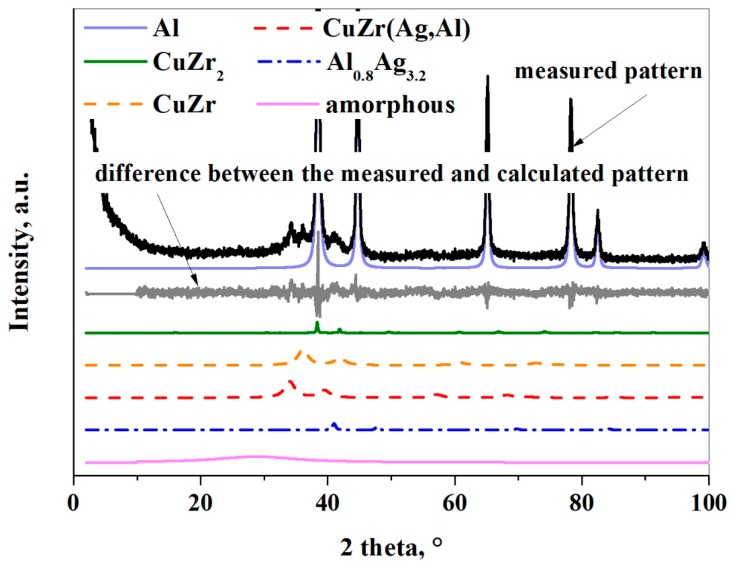
XRD pattern of the composite powder after 30 h of milling with using ethanol.

The initial phases remain, except the α-Fe contamination based on the XRD analysis (Figure 11). However, their weight fraction reduces greatly except for the Al phase. The crystallite sizes are much larger than in the first experiment owing to the ethanol layer. Two different CuZr phases were detected by XRD. The unit cell of the initial CuZr(Ag,Al) phase has changed from 0.4541 nm to 0.4559. It can be explained that some Al atoms (r_Al_ = 0.1432 nm) replace Cu atoms (r_Cu_ = 0.1278 nm). The unit cell of the new CuZr phase is 0.4308 nm, which is closer than the equivalent unit cell of the CuZr phase (a = 0.3262 nm). The α-Fe contamination phase is not detectable after 30 h milling time.

Modeling amorphous components, or “short-range ordered phases”, by the peak fitted to the halo (hump) method was useful and allowed us to extract information on the composition of the amorphous phases and the size range of domains with different compositions. The results show that the presence of PCA is necessary for the formation of an amorphous phase because the ‘X-ray amorphous’ fraction increases by more than nine times after 30 h of milling (Table 1). The maximum position of the amorphous halo has increased from 0.2182 nm to 0.3203 nm after 30 h milling time. The maximum position of the halo reflects the most frequent distances between atoms, so the amorphous structure contains larger atoms than in the case of the raw material. In this system, the larger atom is Zr with a 0.1603 nm atomic radius, while Ag, Fe, and Cu are smaller atoms having atomic radii of 0.1445, 0.1241, and 0.1278 nm, respectively. The amount of the initial Zr containing crystalline phases as CuZr_2_, CuZr(Ag,Al) decreased drastically, so the amorphous phase is enriched by zirconium.

**Figure 12:**
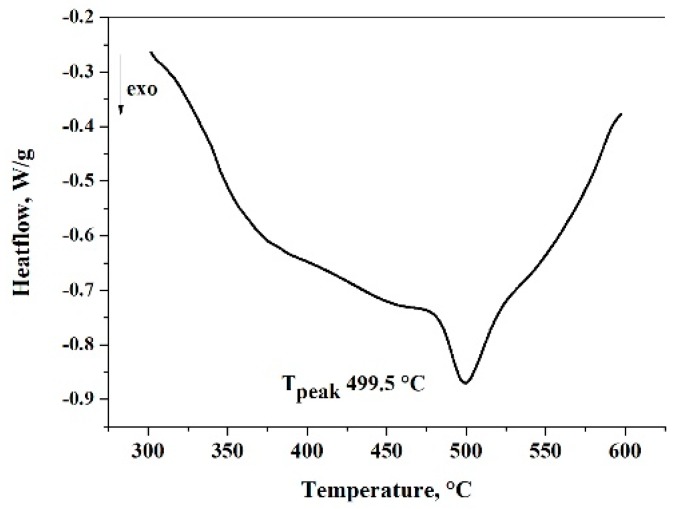
DSC curve from the composite powder after 30 h milling time using ethanol measured at a heating rate of 40 K/min.

The thermal analysis of composite powder confirms the presence of the amorphous phase. The peak position of crystallization changes from 512 °C to 499.5 °C, which means that the composition of the amorphous phase has changed (Figure 12).

**Figure 13:**
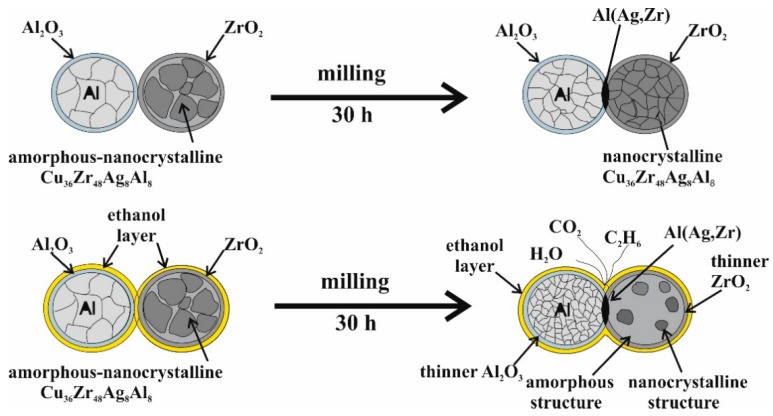
Schematic of the milling process without and with ethanol.

It is known from the literature that organic PCAs decompose during milling [22,23]. Some authors reported that oxygen, carbon, and hydrogen interact with the milled powder. Al_2_O_3_ and Al_4_C_3_ phases were revealed as impurities in the structure of the material [36,37], but both phases were not detectable after 30 h milling time in the present study. In order to clarify the effect of ethanol during the milling process and knowing that ethanol has a reduction ability and that the input energy is very high during the ball milling process, the volatile substances in the milling bowl were analyzed by gas chromatography. A schematic of the milling process without and with ethanol is shown in Figure 13.

**Figure 14:**
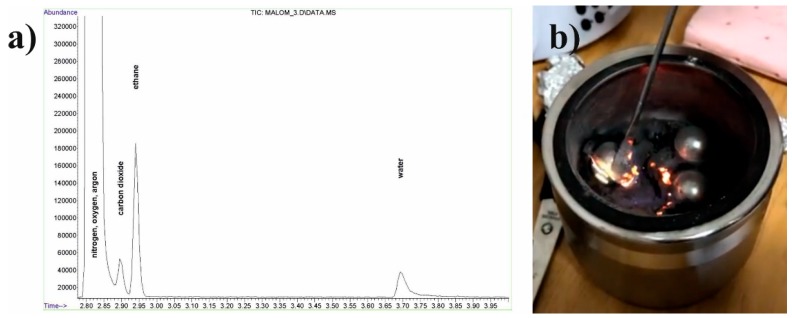
(**a**) Chromatogram of volatile substances in the milling bowl and (**b**) burning of ethane after 30 h milling time.

Figure 14a confirms that ethanol has decomposed to carbon dioxide, water, and ethane. It is explained by the involvement of oxygen from the oxide layer of the particles and of hydrogen dissolved in the alloy. The first step of the reaction could be the dehydration of ethanol (Equation (1)). At the same time, the hydrogenation of ethylene to ethane (Equation (2)) and the oxidation of ethanol (Equation (3)) takes place in the system. All the necessary conditions, such as high temperature and catalysts (Al_2_O_3_ from the oxide layer and Ni from the balls and bowl) are provided in the course of milling. A small hydrogen content can be found in the amorphous-nanocrystalline Cu_36_Zr_48_Ag_8_Al_8_ alloy which can interact with ethylene when the particles break, and a new active layer develops.

(1) $$C_{2}H_{5}-\mathrm{OH}\overset{t{}^{\circ}C,{\mathrm{Al}}_{2}O_{3}}{\to }{\mathrm{CH}}_{2}={\mathrm{CH}}_{2}+H_{2}O$$

(2) $${\mathrm{CH}}_{2}={\mathrm{CH}}_{2}+H_{2}\overset{t{}^{\circ}C,\mathrm{Ni}}{\to }{\mathrm{CH}}_{3}-{\mathrm{CH}}_{3}$$

(3) $$C_{2}H_{5}-\mathrm{OH}+3O_{2}\overset{t{}^{\circ}C}{\to }2{\mathrm{CO}}_{2}+3H_{2}O$$

Figure 14b confirms the presence of combustible gas by burning as soon as the grinding bowl is opened. The oxygen atoms for the oxidation of ethanol are obtained from the oxide layer of the surface of the aluminum and Cu_36_Zr_48_Ag_8_Al_8_ powder. As a result, ethanol decreases the oxygen content of the milling material, which helps develop the amorphous content. Comparing the high-energy ball milling without and with ethanol it can be established that ethanol not only reduces the surface tension, but the abovementioned (Equations (1)–(3)) mechanochemical processes promote the development of the amorphous-nanocrystalline structure.

## 4. Conclusions

Pure Al and amorphous-nanocrystalline Cu_36_Zr_48_Ag_8_Al_8_ alloy powder were milled with and without ethanol by a high-energy mill for up to 30 h. The main conclusions which can be drawn are as follows:

In the case of dry milling:

1. The amorphous phase disappears after 5 h milling time and does not develop until 30 h milling time. The thin oxide layer can act as a heterogeneous nucleation site for crystallization.
2. The repetitive impact of the balls can create a strong bond between the Al-Al particles and Al-reinforcing particles after 15 h milling time.
3. The most significant change in the crystallite size takes place between 0 and 15 h of milling time.

Milling the Al powder for the composite matrix leads to a considerable refinement; the grain size of the Al matrix decreases to 113 nm. The crystallite size of the reinforcing phases is between 1.27 and 10.9 nm after 30 h of milling.

In the case of wet milling:

1. Ethanol as PCA not only decomposes during high-energy milling as it is known from the literature. Ethylene is formed during the dehydration of ethanol, and at the same time, the hydrogenation of ethylene to ethane and the oxidation of ethanol takes place in the system.
2. Ethanol decomposes to carbon dioxide, water, and ethane during high-energy milling because all the necessary conditions, such as high temperature and catalysts (Al2O3 from the oxide layer, Ni from the balls and bowl, and hydrogen from the Cu-Zr based particles) are provided in the course of Al milling with Cu36Zr48Ag8Al8 alloy powder milling. The amorphous-nanocrystalline structure is formed thanks to these mechanochemical processes.
3. The amount of the amorphous phase increases from 0.1 to 10 wt.% after 30 h of milling owing to the reduction of the oxide layer by ethanol.
4. The crystallite size of the Al matrix is between 29 and 46 nm after 30 h of milling time. The crystallite size of the reinforcing phases varies from 5–200 nm.

## Figures and Tables

**Table 1:** Features of the amorphous phase and the crystallites based on the XRD of raw and milled powder.

Phase	Milling Time, h	Without Ethanol							With Ethanol
		0	5	10	15	20	25	30	30
**Al** Fm_3m	crystallite size, nm	494–776	188–296	50–79	70–110	55–86	57–90	72–113	29–46
	wt. % Rietveld	90.00	89.43	86.13	82.24	85.77	84.46	83.84	87.68
**CuZr_2_** I4/mmm	crystallite size, nm	1.3–2.0	1.27–2.0	1.27–2.0	1.27–2.0	1.27–2.0	1.27–2.0	1.27–2.0	31–56
	wt. % Rietveld	7.066	6.50	6.59	8.61	6.73	6.86	7.46	0.28
**CuZr(Ag,Al)** Fm_3m	crystallite size, nm	1–2.21	1.27–2.0	1.27–2.0	1.27–2.0	1.27–2.0	1.27–2.0	1.27–2.0	3–5
	wt. % Rietveld	0.739	0.95	1.51	1.92	1.73	1.97	2.10	0.35
**CuZr** Fm_3m	crystallite size, nm	–	–	–	–	–	–	–	3–5
	wt. % Rietveld	–	–	–	–	–	–	–	0.34
**Al_0.8_Ag_3.2_** Fm_3m	crystallite size, nm	2.0–3.3	42–66	5.9–9.3	9.1–14.3	5.4–8.4	4.6–7.3	6.7–10.5	6–10
	wt. % Rietveld	0.845	2.11	4.64	5.17	4.48	5.12	4.93	0.35
**-Iron** Im_3m	crystallite size, nm	20.6–32.4	3.6–5.8	7.6–12.1	5.4–8.4	6.7–10.5	7.9–12.5	6.9–10.9	–
	wt. % Rietveld	0.150	1.02	1.13	2.07	1.29	1.59	1.67	–
**amorphous**	wt. % Rietveld	0.12	0	0	0	0	0	0	11.00
